# SPNS2 Downregulation Induces EMT and Promotes Colorectal Cancer Metastasis *via* Activating AKT Signaling Pathway

**DOI:** 10.3389/fonc.2021.682773

**Published:** 2021-06-24

**Authors:** Lei Lv, Qiyi Yi, Ying Yan, Fengmei Chao, Ming Li

**Affiliations:** ^1^ Department of Cancer Epigenetics Program, Anhui Provincial Cancer Hospital, The First Affiliated Hospital of USTC, Division of Life Sciences and Medicine, University of Science and Technology of China, Hefei, China; ^2^ School of Basic Medical Sciences, Anhui Medical University, Hefei, China; ^3^ Department of Oncology, The First Affiliated Hospital of USTC, Division of Life Sciences and Medicine, University of Science and Technology of China, Hefei, China; ^4^ Department of Laboratory Medicine, The First Affiliated Hospital of USTC, Division of Life Sciences and Medicine, University of Science and Technology of China, Hefei, China

**Keywords:** SPNS2, colorectal cancer (CRC), PTEN/AKT, invasion, metastasis, epithelial–mesenchymal transition

## Abstract

Spinster homologue 2 (SPNS2), a transporter of S1P (sphingosine-1-phosphate), has been reported to mediate immune response, vascular development, and pathologic processes of diseases such as cancer *via* S1P signaling pathways. However, its biological functions and expression profile in colorectal cancer (CRC) is elusive. In this study, we disclosed that SPNS2 expression, which was regulated by copy number variation and DNA methylation of its promoter, was dramatically upregulated in colon adenoma and CRC compared to normal tissues. However, its expression was lower in CRC than in colon adenoma, and low expression of SPN2 correlated with advanced T/M/N stage and poor prognosis in CRC. Ectopic expression of SPNS2 inhibited cell proliferation, migration, epithelial–mesenchymal transition (EMT), invasion, and metastasis in CRC cell lines, while silencing SPNS2 had the opposite effects. Meanwhile, measuring the intracellular and extracellular level of S1P after overexpression of SPNS2 pinpointed a S1P-independent model of SPNS2. Mechanically, SPNS2 led to PTEN upregulation and inactivation of Akt. Moreover, AKT inhibitor (MK2206) abrogated SPNS2 knockdown-induced promoting effects on the migration and invasion, while AKT activator (SC79) reversed the repression of migration and invasion by SPNS2 overexpression in CRC cells, confirming the pivotal role of AKT for SPNS2’s function. Collectively, our study demonstrated the suppressor role of SPNS2 during CRC metastasis, providing new insights into the pathology and molecular mechanisms of CRC progression.

## Introduction

Colorectal cancer (CRC) is the third most common tumor all over the world, with nearly two million patients have been diagnosed with colorectal cancer in 2020, and it is also the second highest cause of cancer-associated mortalities, with 935,000 deaths in 2020 globally (1). The vast majority of CRC-related mortality is attributed to metastasis, which is considered to be the most difficult challenge for CRC treatment. The five-year relative survival rate is 90% for patients diagnosed with localized disease, but decreases to 71% and 14% for those diagnosed with regional and distant stages, respectively (2). Despite significant developments of therapeutic strategies, effective therapy for CRC with metastasis is deficient. Targeting cancer cells with high invasion/metastasis potential from the primary site may be a promising field in anti-metastasis therapy. Thus, understanding the specific mechanism of CRC progression, especially how CRC acquire metastatic properties, is important for the development of the diagnostic techniques and therapeutic strategies for CRC patients.

Other than peptides and proteins, recent research have shown that lipids and lipids metabolism were also linked to metastasis (3–6). Sphingosine 1-phosphate (S1P), one kind of bioactive lipid, takes part in many cellular processes, including proliferation, apoptosis, migration, invasion and angiogenesis in various tumors (7–10). After being produced by SPHK1 and SPHK2, both of which are sphingosine kinases in cells, it can interact with specific intracellular proteins, such as TRAF2, cIAP2 and hTERT, to regulate cellular responses (11–13). It also can be exported out of the cells, where it binds to S1P1-5, which are S1P-specific G protein coupled receptors to simulate downstream signals and execute its function. There are several transporters of S1P, including SPNS2, ABCC1 and ABCG2. SPNS2, a non-ATP dependent transporter, transports S1P from endothelial and lymph-endothelial cells, and regulates S1P concentration in plasma and lymph (14). Besides, it is also involved in oncogenesis and cancer progression, having both positive and negative effects (15). Deficiency of *Spns2* in endothelium inhibited the pulmonary metastasis of melanoma cells through enhancing immune-mediated cell killing by natural killer cells and T cells in mouse model (6). On the contrary, SPNS2 induced apoptosis and inhibited migration ability of NSCLC (non-small cell lung cancer) cells (16). Thus, the effect of SPNS2 on cancer progression is complicated and may be dependent on the types of cancer and microenvironments.

In the present study, we investigated the clinical relevance and specific mechanisms of SPNS2 in CRC. Our clinic-pathological study showed that SPNS2 expression in CRC was frequently lower than in colon adenomas, which were precursor lesions of CRC. Low expression of SPNS2 correlates with advanced CRC stages and poor prognosis of CRC patients. Lack of SPNS2 expression contributed to CRC cell proliferation, migration, invasion and metastasis, possibly through inhibiting PTEN expression and activating Akt signaling pathway. Thus, our findings may shed light on the role of SPNS2 in the CRC progression.

## Materials and Methods

### Data Acquisition in TCGA COADREAD

All CRC clinical data, copy number, DNA methylation, and RNA sequencing data in TCGA COADREAD were retrieved through the UCSC XENA (https://xenabrowser.net) (17). For CNV data, we used the copy number segments after remove germline CNV. For DNA methylation data, we used DNA methylation profile of HumanMethylation450 platform. For RNA sequencing data, we used the pancan normalized gene expression RNAseq data. In addition, TIMER (https://cistrome.shinyapps.io/timer) was used to explore the differential expression between tumor and adjacent noncancerous samples for SPNS2 across all TCGA tumors (18).

### Gene Expression Omnibus Analysis

We systematically searched for colorectal cancer datasets that were publicly available and reported clinical annotations in GEO (Gene Expression Omnibus) and downloaded the data. GEO datasets, including GSE4183 (19), GSE89076 (20), GSE41657, GSE34472, GSE57965 (21), GSE17536 (22), GSE42284 (23) and GSE62322 (24), were used in this study.

### The Kaplan-Meier Plotter

To analyze the correlation of SPNS2 expression and CRC prognosis, including OS (Overall survival), DSS (Disease specific survival), PFI (Progression free interval) and DFS (Disease free survival), the samples were split into high and low expression group and assessed by a Kaplan-Meier survival plot using R package.

### Cell Culture and Reagents

HT-29, HCT116 and SW480 cell lines were purchased from Shanghai Jinfu (Shanghai, China) and cultured in DMEM medium plus 10% FBS at 37°C in 5% CO_2_. For Akt inhibition or activation, 10 μM of MK-2206 (Selleck, S1078) or 10 μg/ml SC79 (Selleck, S7863) were used to treat CRC cells for 48 hours, respectively.

### Silencing and Overexpressing of SPNS2

SPNS2 siRNA and the scramble sequence control (NC) as well as riboFECT CP transfection kit (cat. no. C10511-05) were supplied by Ribobio (Guangzhou, China). The cell transfection was performed according to the manufacturer’s instructions. The siRNA sequences used for SPNS2 interference in this study were as follows (5’→3’): GCCCAAGUUGUGCAGAAGA dTdT and dTdT UCUUCUGCACAACUUGGGC. The SPNS2-overexpression lentivirus (OE-SPNS2) was constructed and packaged by HANBIO (Shanghai, China). For lentivirus transduction, HCT116 cells that reached 50% confluency were transduced with the lentivirus in the presence of 8 μg/ml polybrene and stably infected cells were selected with 2 μg/ml puromycin.

### Cell Proliferation Assay

Cells proliferated at a log phase were seeded in 96-well plates at a density of 2 × 10^3^/well (in triplicate) to allow adhesion. After cultivation for 0, 24, 48, and 72 h later, cells were incubated with 10 µl CCK-8 for an extra 2h. The cell viability was determined by measuring the optical density at 450 nm wavelength using a microplate reader (Tecan Group Ltd.) at 450 nm. Then the cells were cultured with fresh medium until the next round of measurement. The mean and S.D. of the triplet’s measurements were calculated and plotted.

### Transwell Assay and Invasion Assay

Migration and Matrigel invasion assays were performed using 24-well insert, 8 μm pore size with or without pre-coated matrigel from Corning Inc., according to the manufacturer’s directions. Briefly, 1 × 10^5^ cells in 200 μL FBS-free DMEM medium were seeded into the upper portion of the chamber, while 900 μL DMEM medium plus 30% FBS was loaded into the lower side, which served as a chemo-attractant. After 48 h, the non-invasive cells were removed from the upper surface of the membrane with a cotton swab, cells penetrated to the underside of the membrane were fixed and stained with 0.1% crystal violet, and further counted in four random fields under a microscope.

### Pathway Activity Assay

Cells were seeded into 96-well plates at a density of 1×10^4^ cells/well and cultured overnight. Next day, they were transfected with a mixture of AKT, ERK, IL-6 or NF-κB firefly luciferase reporter and the Renilla luciferase construct (Qiagen), with the Attractene transfection reagents (Qiagen) according to the manufacturer’s instruction, both luciferase activities in cell extracts at 24h after transfection were measured by the Dual-Luciferase Reporter Assay System (cat. no. E1910; Promega) using a Promega GloMax 20/20 luminometer. The relative firefly luciferase activities of the pathway reporter constructs were analyzed as previously reported (25).

The pathway activity assay is based on an inducible transcription factor responsive construct. This construct encodes the firefly luciferase reporter gene under the control of a basal promoter element (TATA box) joined to tandem repeats of the cognate consensus motif, which is recognized by each master transcription factor for the corresponding pathway. It monitors both increases and decreases in the activity of a key transcription factor, which is a downstream target of a specific signaling pathway. Specifically, mammalian FOXO protein (FOXO1, FOXO3, FOXO4), a subgroup of Forkhead transcription factors, is among the best characterized targets of the PI3K/AKT signaling pathway (26, 27). Thus, the increase or decrease in the activity of PI3K/Akt pathway can be indicated by the change of the FOXO luciferase activity. In the same way, the activity of Elk-1/SRF, STAT3 and NF-κB corresponds to ERK, IL-6 and NF-κB downstream signaling pathway respectively.

### Reverse Transcription−Quantitative PCR (RT−qPCR)

Total RNA was extracted from the cells with TRNzol-A^+^ reagent (Tiangen Biotech), then was reversed transcribed into cDNA using the HiScript^®^ II 1st Strand cDNA Synthesis Kit (cat. no. R211-01; Vazyme). The RNA level was quantified using the AceQ qPCR SYBR Green Master Mix (cat. no. Q131-02; Vazyme) on a FTC-3000P PCR instrument (FUNGLYN BIOTECH INC, Canada). Using the 2^-ΔΔCt^ method, the relative expression level of target gene was calculated and normalized by GAPDH expression. The sequences of the primers were listed as follows (5’→3’): SPNS2 forward, TGCTTTACGGGATTTCTGGG, and reverse, GGCTCCTACGATGCTGCTCT; GAPDH forward, GGAGCGAGATCCCTCCAAAAT, and reverse, GGCTGTTGTCATACTTCTCATGG.

### Western Blot Analysis

Lysates from CRC cells were separated on 10% SDS polyacrylamide gels, and the proteins were then transferred to a PVDF membrane (cat. no. IPVH00010; Millipore). After blocking with TBST (0.5% Tween-20 in Tris-buffered saline) containing 5% non-fat milk for 1 h at room temperature, the membranes were incubated with primary antibody overnight at 4°C and then with secondary antibodies for 1 h at room temperature. The target bands were revealed by SuperSignal West Pico PLUS chemiluminescence substrate (cat. no. 34580; Thermo Fisher Scientific), and loading differences were normalized to a monoclonal GAPDH antibody. Primary antibodies used in the present study are as follow: SPNS2(1:500, Novus #NBP1-54345), p-AKT(1:1000, CST #4060), AKT (1:1000, CST #C6717), p-ERK1/2 (1:1000, CST #4370), ERK1/2 (1:1000, CST #4695), p-p65 (1:1000, Immunoway #YD0191), p65 (1:1000, proteintech #10745-1-AP), p-JNK (1:1000, CST #9255), JNK (1:1000, CST #9252), and GAPDH (1:2000, proteintech # 60004-1-Ig). HRP-conjugated Goat Anti-Rabbit IgG(H+L) (1:5000, proteintech #SA00001-2) and HRP-conjugated Goat Anti-Mouse IgG(H+L) (1:5000, proteintech #SA00001-1) are served as secondary antibodies.

### BSP Analysis

Genomic DNA was extracted from cells by PureGenome™ Kit (cat. no. P-9040-M; Aline Bioscience) and qualified by electrophoresis on an agarose gel. The bisulfite conversion was achieved by EZ DNA Methylation-Gold Kit (cat. no. D5006; ZYMO Research). Part of the CpG island upstream of SPNS2 gene was amplified by the primers listed below (5’→3’): forward, GATTAGGATGGTGTAGTGGYG and reverse, CATTCCAAACACATCATACCRAC. The PCR products from bisulfite treated DNA were cloned and verified by sequencing as described previously (28).

### Aza-2’-Deoxycytidine Treatment

HCT116 cells were treated with 50 mM 5-aza-2’-deoxycytidine for 96 hours with a change of culture medium every 24 hours as previously described (29).

### S1P Measurement

Cells were grown for 48 hours and then both cell pellets and media were collected. Conditioned medium was collected and centrifuged at 12,000 g for 5 minutes at 4°C to remove cell debris. Cells were detached and pellets were washed three times with cold PBS. S1P levels in cells and culture supernatant were measured by liquid chromatography-tandem mass spectrometry (LC-MS/MS) as described previously (30).

### *In Vivo* Mouse Metastasis Assay

Animal experiments were undertaken in accordance with the National Institutes of Health Guide for the Care and Use of Laboratory Animals. 4×10^6^ HCT116 OE-NC or HCT116 OE-SPNS2 cells suspended in 100μl PBS were injected into male athymic nude mice aged 4-5 weeks through the tail vein. 10 weeks after injection, all mice were sacrificed, tissue from lung was excised, and the number of metastatic nodules formed was counted and analyzed by hematoxylin and eosin (HE) staining.

### Statistical Analysis

Data are presented as means, and error bars indicate the standard deviation (S.D.). All statistical analyses were performed with Prism (GraphPad Software Inc., La Jolla, CA, USA). Two way anova and two-tailed Student’s *t*-test were used to calculate statistical significance. A P-value of <0.05 was considered to be significant.

## Results

### Low SPNS2 Levels Are Associated With Worse Clinic-Pathological Parameters and Prognosis in Colorectal Cancer

**Figure 1:**
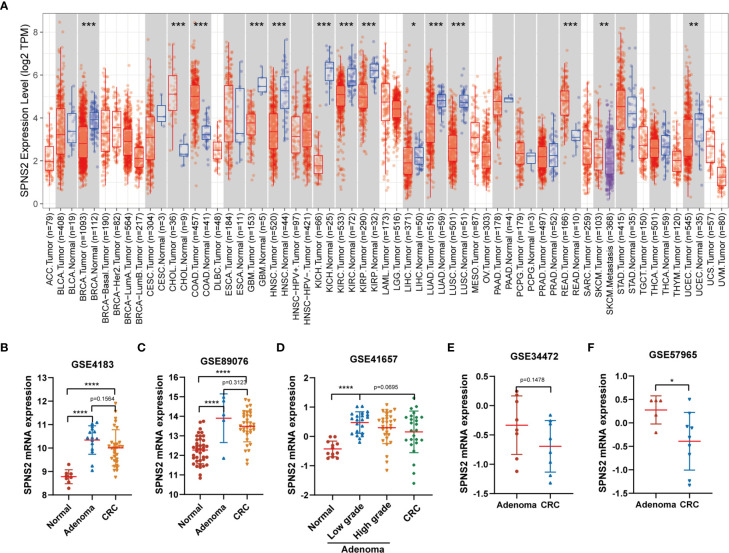
SPNS2 mRNA expression in human cancer and noncancerous tissues. **(A)** SPNS2 expression levels in tumor and adjacent noncancerous tissue from TCGA database. Comparison of SPNS2 expression in tumors and corresponding normal tissues in different types of cancer, which were displayed in gray columns when normal data are available. (Red indicates tumor samples; blue indicates noncancerous samples; purple indicates tumor samples with metastasis; The number of samples in each group is represented in parentheses). ACC, Adrenocortical carcinoma; BLCA, Bladder Urothelial Carcinoma; BRCA, Breast invasive carcinoma; CESC, Cervical squamous cell carcinoma and endocervical adenocarcinoma; CHOL, Cholangio carcinoma; COAD, Colon adenocarcinoma; DLBC, Lymphoid Neoplasm Diffuse Large B-cell Lymphoma; ESCA, Esophageal carcinoma; GBM, Glioblastoma multiforme; HNSC, Head and Neck squamous cell carcinoma; KICH, Kidney Chromophobe; KIRC, Kidney renal clear cell carcinoma; KIRP, Kidney renal papillary cell carcinoma; LAML, Acute Myeloid Leukemia; LGG, Brain Lower Grade Glioma; LIHC, Liver hepatocellular carcinoma; LUAD, Lung adenocarcinoma; LUSC, Lung squamous cell carcinoma; MESO, Mesothelioma; OV, Ovarian serous cystadenocarcinoma; PAAD, Pancreatic adenocarcinoma; PCPG, Pheochromocytoma and Paraganglioma; PRAD, Prostate adenocarcinoma; READ, Rectum adenocarcinoma; SARC, Sarcoma; SKCM, Skin Cutaneous Melanoma; STAD, Stomach adenocarcinoma; TGCT, Testicular Germ Cell Tumors; THCA, Thyroid carcinoma; THYM, Thymoma; UCEC, Uterine Corpus Endometrial Carcinoma; UCS, Uterine Carcinosarcoma; UVM, Uveal Melanoma. **(B–F)** Human SPNS2 mRNA expression in CRC, adjacent noncancerous tissue and colon adenoma (including low-grade dysplasia and high-grade dysplasia) from various GEO datasets. ****p < 0.0001, ***p < 0.001, **p < 0.01, *p < 0.05.

To explore the potential role and specific mechanism of SPNS2 in cancer progression, we assessed the expression profiles of SPNS2 in tumor and adjacent noncancerous tissues in TCGA datasets using the “DiffExp module” of TIMER (18). SPNS2 expression was markedly lower in ten kinds of tumor compared to the adjacent noncancerous samples, such as BRCA (Breast invasive carcinoma), LIHC (Liver hepatocellular carcinoma), LUAD (Lung adenocarcinoma) and LUSC (Lung squamous cell carcinoma) (**Figure 1A**). Moreover, its expression in metastasis SKCM (Skin Cutaneous Melanoma) was significantly lower than in primary SKCM (**Figure 1A**), suggesting a general tumor suppressor role of SPNS2. However, its expression in CHOL (Cholangio carcinoma), COAD (Colon adenocarcinoma) and READ (Rectum adenocarcinoma) were higher than in adjacent noncancerous tissues (**Figure 1A**). The higher expression of SPNS2 in CRC was further confirmed in three CRC datasets, including GSE4183, GSE89076 and GSE41657, from GEO (Gene Expression Omnibus) (**Figures 1B–D**). However, we found that SPNS2 expression in CRC was lower than that in colon adenomas, which are precursor lesions of CRC, from analysis CRC datasets GSE4183, GSE89076, GSE41657, GSE34472 and GSE57965 (**Figures 1B–F**).

**Figure 2:**
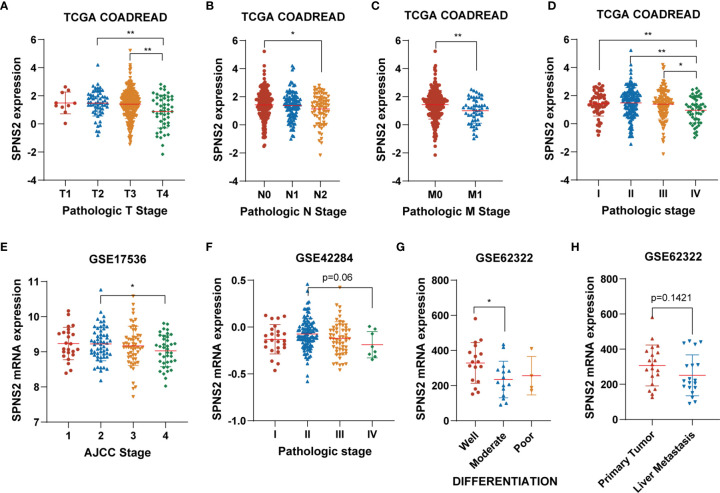
The association between SPNS2 expression and clinic-pathological parameters of CRC patients. **(A–D)** SPNS2 expression levels in different T, N, M and pathologic stages in TCGA COADREAD dataset. **(E)** SPNS2 expression levels in different AJCC stages in GSE17536 dataset. **(F)** SPNS2 expression levels in different pathologic stages in GSE42284 dataset. **(G)** SPNS2 expression levels in different differentiation status in GSE62322 dataset. **(H)** SPNS2 expression in primary tumor and colon tumor with liver metastasis in GSE62322 dataset. **p < 0.01, *p < 0.05.

To further explore the impact of SPNS2 on CRC progression, we then analyzed the correlation between SPNS2 expression and the clinic pathological parameters of CRC patients in TCGA CRC datasets, including COAD (Colon adenocarcinoma) and READ (Rectum adenocarcinoma). We found that low expression of SPNS2 was associated with advanced T, M, N, and pathologic stage (**Figures 2A–D**). Analysis from GSE17536, GSE42284 and GSE62322 also showed that the SPNS2 expression was significantly lower in samples from high AJCC (The American Joint Committee on Cancer) stage, pathologic stage and grade (**Figures 2E–G**). And its expression in CRC tumor with liver metastasis was lower than in primary tumor (**Figure 2H**).

**Figure 3:**
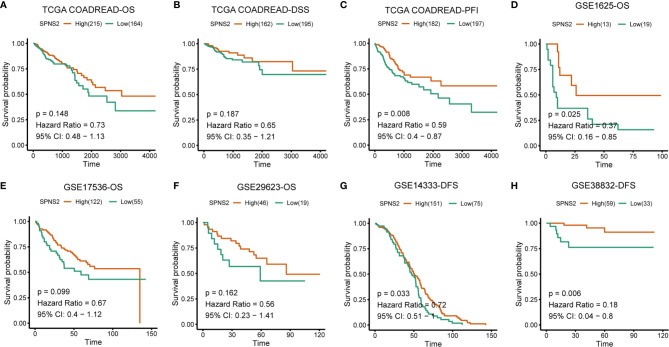
Low SPNS2 mRNA expression predicts poor survival rates of CRC patients. **(A–C)** Kaplan-Meier analysis of OS, DSS and PFI in the TCGA COADREAD patients based on SPNS2 expression. **(D–F)** Kaplan-Meier analysis of OS in the patients with CRC based on SPNS2 expression from GSE1625, GSE17536 and GSE29623. **(G–H)** Kaplan-Meier analysis of DFS in the patients with CRC based on SPNS2 expression from GSE14333 and GSE38832. Red line: high SPNS2 expression group; Green line: low SPNS2 expression group. Log-rank P values were used to compare curves between subgroups. OS, Overall survival; DSS, Disease specific survival; PFI, Progression-free interval; DFS, Disease-free survival.

At last, the prognostic value of SPNS2 expression in CRC was evaluated. Kaplan-Meier Plotter analysis showed that low SPNS2 expression significantly correlated with shorter PFI (Progression-free interval) (p = 0.008), and also correlated with shorter OS (overall survival) (p = 0.148), DSS (Disease-specific survival) (p = 0.187) in TCGA COADREAD (**Figures 3A–C**), indicating a worse prognosis in patients with lower SPNS2 expression. Worse OS (overall survival) was also observed in CRC patients with lower SPNS2 expression in GEO CRC datasets, including GSE1625 (p=0.025), GSE17536 (p=0.099) and GSE29623 (p=0.162) (**Figures 3D–F**). Worse DFS (Disease-free survival) was observed in CRC patients with lower SPNS2 expression in GSE14333 (p=0.033) and GSE38832 (p=0.006) (**Figures 3G, H**).

Collectively, these results suggested that SPNS2 might promote tumorigenesis in the early stage, but inhibit tumor progression in the late stage of CRC, and its expression is a prognostic factor for colorectal cancer.

### Regulation of SPNS2 Expression in Colorectal Cancer

Subsequently, we examined how expression of the SPNS2 is regulated in CRC. Methylation change and copy number alteration (CNA) are classical transcriptional regulator of gene expression. We then investigated whether the SPNS2expression was regulated by these two factors.

**Figure 4:**
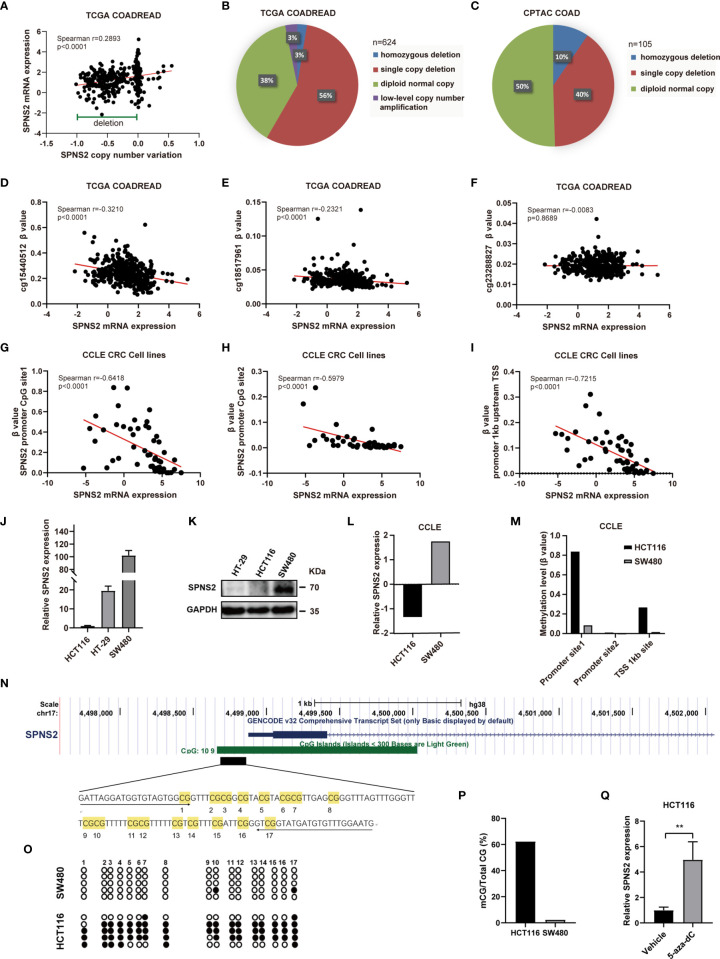
Expression of SPNS2 is regulated by copy number variation (CNV) and DNA methylation. **(A)** Correlation analysis of SPNS2 expression and copy number performed in TCGA-COADREAD dataset. **(B)** The proportion of samples with different copy number alterations of SPNS2 in TCGA COADREAD. **(C)** The proportion of samples with different copy number alterations of SPNS2 in CPTAC COAD. **(D–F)** Spearman’s correlation analysis of SPNS2 expression and β value of cg15440512, cg18517961 and cg23288827 performed in TCGA-COADREAD dataset. **(G–I)** Scatter plot of Spearman’s correlation analysis of SPNS2 expression and β value of CpG sites performed in CCLE CRC cell lines. **(J)** mRNA levels of SPNS2 were determined by quantitative RT-PCR in HCT116, HT29 and SW480 cells. **(K)** Protein levels of SPNS2 were estimated by western blotting in HCT116, HT29 and SW480 cells. **(L)** SPNS2 mRNA levels of SW480 and HCT116 cells in CCLE. **(M)** Methylation level of SPNS2 promoter of HCT116 and SW480 cells in CCLE. **(N)** A schematic representing the location of SPNS2 in the genome using the UCSC browser. A 122−bp region CpG island containing 17 CpGs analyzed by BSP are indicated. **(O)** DNA methylation state of the SPNS2 gene in HCT116 and SW480 cells was determined by BSP. The unmethylated CpGs are indicated with open circles, and the methylated CpGs are indicated with filled circles. **(P)** All the data of methylation in **(O)** are summarized. **(Q)** Effect of 5-aza on the SPNS2 mRNA expression in HCT116 cells. **P < 0.01.

To detect whether SPNS2 CNA contributes to SPNS2 dysregulation or not, we extracted the CNA and expression data of SPNS2 from TCGA COADREAD. We evaluated the correlation between the SPNS2 expression and its copy number by Spearman correlation coefficient, which showed that SPNS2 expression positively correlated with its copy number in TCGA COADREAD (Spearman r = 0.2893, P <0.0001; **Figure 4A**). SPNS2 copy number was frequently deleted in CRC tissues of TCGA COADREAD cohort (**Figures 4A, B**), 3% of which were homozygous deletion and 56% were single copy deletion. Similarly, 10% of CRC samples were homozygous deletion and 40% were single copy deletion in CPTAC (Clinical Proteomic Tumor Analysis Consortium) COAD dataset (**Figure 4C**). These results demonstrated that downregulation of SPNS2 expression was partially due to copy number deletion in CRC.

DNA methylation often negatively regulating gene expression through an epigenetic mechanism. After analyzing the methylation alterations of CpG loci in the promoter of SPNS2 gene in TCGA COADREAD, methylation level (β value) of two methylated loci, including cg15440512 and cg18517961, were found to be negatively correlated with SPNS2 mRNA expression significantly (r = -0.3210, p<0.0001; r = -0.2321, p < 0.0001 respectively) (**Figures 4D–F**). To confirm our findings in TCGA COADREAD methylation array, SPNS2 mRNA expression and methylation levels of its promoter in CCLE (Cancer Cell Line Encyclopedia) CRC cell lines were also analyzed. Methylation level of CpG loci in SPNS2 gene’s promoter (**Figures 4G, H**) and TSS upstream 1kb (**Figure 4I**) negatively correlated with SPNS2 expression significantly in CRC cell lines (r = -0.6418, p < 0.0001; r = -0.5979, p < 0.0001; r = -0.7215, p < 0.0001; respectively). Furthermore, the negative association between SPNS2 expression and its promotor methylation level was validated by qRT-PCR, WB and BSP analysis in CRC cell lines. The results showed that SPNS2 mRNA level was lowest in HCT116 cells, and highest in SW480 cells (**Figure 4J**), which was consistent with analysis from CCLE (**Figure 4L**). The protein level of SPNS2 in SW480 was also significantly higher than in HT29 and HCT116 (**Figure 4K**). There is a CpG island (CGI) containing 109 CpG sites in SPNS2 promoter and the first exon (**Figure 4N**). A bisulfite conversion sequencing (BSP) analysis amplifying the 17 CpG sites in this region revealed that they were hypermethylated in HCT116, but barely methylated in SW480 cells (**Figures 4O, P**), which was consistent with analysis from CCLE database (**Figure 4M**). Additionally, the mRNA expression of SPNS2 increased by >4−fold in HCT116 cells after treated with 5-aza-dC, a DNA methylation inhibitor, for three days (**Figure 4Q**).

Taken together, SPNS2 expression is regulated through promoter methylation and copy number variation.

### SPNS2 Inhibits CRC Cell Proliferation

**Figure 5:**
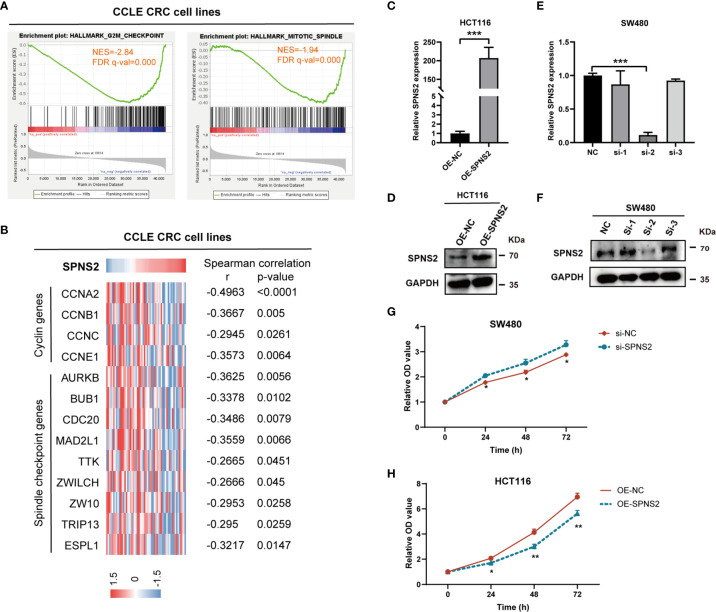
SPNS2 inhibits proliferation of CRC cells. **(A)** GSEA analysis of SPNS2 expression in CCLE CRC cell lines showed that SPNS2 expression negatively correlated with “HALLMARK G2M CHECKPOINT” and “HALLMARK MITOTIC SPINDLE”. NES, normalized enrichment score; FDR, false discovery rate. **(B)** Correlation of SPNS2 mRNA expression with expression of cell cycle related genes in CCLE CRC cells. Expression of cell cycle related genes, including 4 cyclin genes and 9 spindle checkpoint genes, are visualized colorimetrically with heatmap, in which rows represent genes and columns represent CRC cell lines. ‘Red’ representing high gene expression, ‘blue’ representing low gene expression. Statistical analyses were performed by Spearman’s correlation. **(C, D)** The RNA and protein level of SPNS2 determined by qRT-PCR and WB in HCT116 cells overexpressing SPNS2. **(E, F)** siRNA mediated silence of SPNS2 expression in SW480 cells. **(G, H)** CCK8 assay were performed to analyze the effect of SPNS2 overexpression or knockdown on CRC cell proliferation *in vitro*. NC: negative control for siRNA; si: siRNA silencing; OE-NC: negative control for overexpression; OE-SPNS2: SPNS2 overexpression. Statistical analyses were performed by Student’s t-test. ***P < 0.001, **p < 0.01, *p < 0.05.

To explore the functions of SPNS2-mediated CRC progression, we performed GSEA (Gene Set Enrichment Analysis) to profile the positively and negatively correlated genesets with SPNS2 expression based on the RNAseq data from CCLE CRC cell lines. It revealed that SPNS2 expression was negatively associated with the functional gene sets of cell proliferation, including “HALLMARK G2M CHECKPOINT” (NES = -2.84, FDR q-value = 0.000) and “HALLMARK MITOTIC SPINDLES” (NES = -1.94, FDR q-value = 0.000) (**Figure 5A**). Moreover, the expression of cyclins and spindle checkpoint genes negatively correlated with the expression of SPNS2 in CCLE CRC cell lines (**Figure 5B**). Consistently, SPNS2 expression levels were lower in advanced T stages (**Figure 2A**), which describes the size of the main tumor. These results showed that SPNS2 might suppress cell proliferation. Then, the overexpression and loss-of-function studies were performed in CRC cell lines to study the potential role of SPNS2 in CRC tumorigenesis. According to the above results, we chose HCT116 (SPNS2 low expression) and SW480 (SPNS2 high expression) for further experiments. We transfected SW480 cells with the siRNAs targeting SPNS2, and transfected HCT116 cells with SPNS2 expression lentivirus. The mRNA and protein level of SPNS2 substantially increased in SPNS2-expression lentivirus‐transfected HCT116 cells, and significantly reduced in SW480 cells transfected with SPNS2 siRNA-2 (**Figures 5C–F**) compared to the control cells. SPNS2 knockdown enhanced the proliferation of SW480, and SPNS2 overexpression inhibited the proliferation of HCT116 (**Figures 5G, H**), which suggested that SPNS2 impeded the growth of CRC.

### SPNS2 Suppresses Epithelial Mesenchymal Transition, Migration, Invasion and Metastasis in Colorectal Cancer

**Figure 6:**
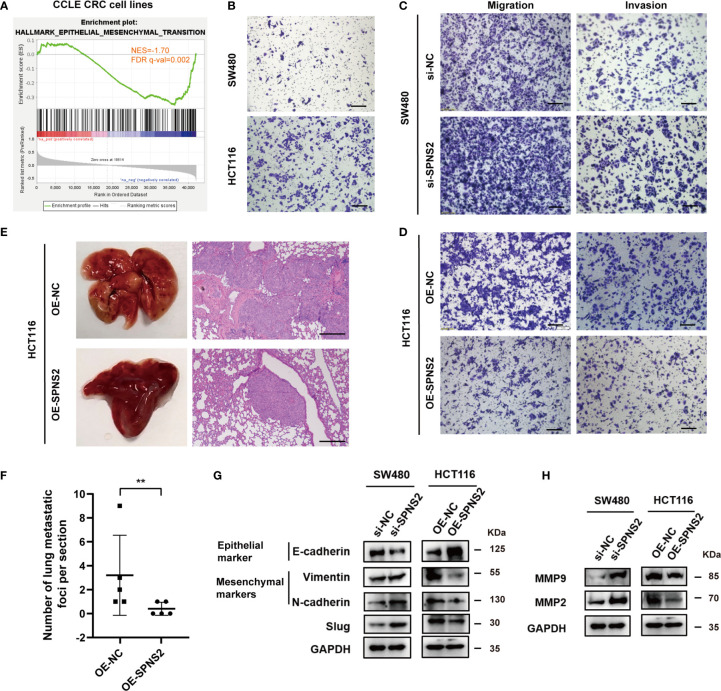
SPNS2 inhibits the cell migration, invasion and metastasis of CRC cells. **(A)** GSEA analysis of SPNS2 expression in CCLE CRC cell lines showed that SPNS2 expression was negatively correlated with “HALLMARK Epithelial–mesenchymal transition”. NES, normalized enrichment score; FDR, false discovery rate. **(B)** Comparison of the invasion ability between SW480 and HCT116 cells through transwell assay. **(C, D)** Transwell assay was performed to analyze the effect of SPNS2 on CRC cell migration and invasion *in vitro*. (scale bar = 100 µm). **(E)** A lung metastasis model of nude mice was generated using HCT116 with SPNS2 overexpression or control (n=5 per group). Left: representative lung tissues, Right: representative HE staining images of the lung tissues (scale bar = 200 µm). **(F)** The number of metastatic nodules on lung surface per mouse from **(E)**. **p < 0.01. **(G)** The expression levels of Slug, E-cadherin, Vimentin and N-cadherin in the indicated cells transfected with si-SPNS2/OE-SEPNS2 or negative control. **(H)** The expression levels of MMP2 and MMP9 in the indicated cells transfected with si-SPNS2/OE-SEPNS2 or negative control. si-NC, negative control for siRNA silencing; si-SPNS2, SPNS2 siRNA silencing; OE-NC, negative control for overexpression; OE-SPNS2, SPNS2 overexpression.

GSEA analysis also revealed that SPNS2 expression was negatively associated with the functional gene sets of “HALLMARK EPITHELIAL MESENCHYMAL TRANSITION” (NES = -1.70, FDR q-value = 0.002, **Figure 6A**) in CCLE CRC cell lines, in line with the above findings that SPNS2 expression levels were lower in advanced N/M stages and liver metastasis (**Figures 2B, C, H**). Then we investigated the effect of SPNS2on migration, invasion and metastasis of CRC cells. Transwell assay showed that the motility of HCT116 which possessed low SPNS2 expression was significantly higher than SW480 cells which had a high level of SPNS2 (**Figure 6B**). Indeed, transwell assay showed that SPNS2 substantially inhibited the migration and invasion of HCT116 cells, while the SPNS2-knockdown enhanced the migration and invasion of SW480 cells (**Figures 6C, D**). To further examine the role of SPNS2 in metastasis *in vivo*, we assessed the metastatic nodules in the lungs in nude mice, which were injected with HCT116 cells into tail vein. Ectopic overexpression of SPNS2 markedly reduced the lung homing potential of HCT116 cells (**Figures 6E, F**). These observations demonstrated that SPNS2 strongly inhibited migratory and invasive capacities of CRC.

EMT (Epithelial-mesenchymal transition) is essential for tumor invasion and metastasis (31). During this progress, cells lose their epithelial traits and acquire mesenchymal traits. We then evaluated the effect of SPNS2 expression on the key components involved in EMT. The protein levels of the E-cadherin (epithelial marker) increased, accompanied by the decrease of vimentin and N-cadherin (mesenchymal markers) in SPNS2-overexpressing HCT116 cells (**Figure 6G**). While in SW480 cells, E-cadherin was downregulated, N-cadherin and vimentin were upregulated after SPNS2 knockdown. Similarly, the mRNA expression of CDH1, which encode the E-cadherin protein, was positively correlated with mRNA expression of SPNS2 (**Figure S1A, D**), while the mRNA expression of CDH2 and VIM, which encode the N-cadherin and vimentin protein, negatively correlated with mRNA expression of SPNS2 in CCLE cell lines (**Figures S1B, C, E, F**). By screening EMT-related transcription factors, we found that SPNS2 markedly inhibited Slug protein expression (**Figure 6G**). These results indicated that SPNS2 inhibited EMT in CRC. In addition, silencing of SPNS2 increased MMP2 and MMP9 expression, while overexpression of SPNS2 decreased the MMP2 and MMP9 expression (**Figure 6H**), which could degrade the extracellular matrix and stimulate tumor invasion and metastasis (32). Accordantly, the SPNS2 mRNA expression negatively correlated with MMP2 and MMP9 mRNA expression in CCLE cell lines (**Figures S1G–J**).

Collectively, these results indicated that loss of SPNS2 could promote migration, invasion, metastasis and EMT in CRC.

### SPNS2 Inactivates PI3K/Akt Signaling in Colorectal Cancer

**Figure 7:**
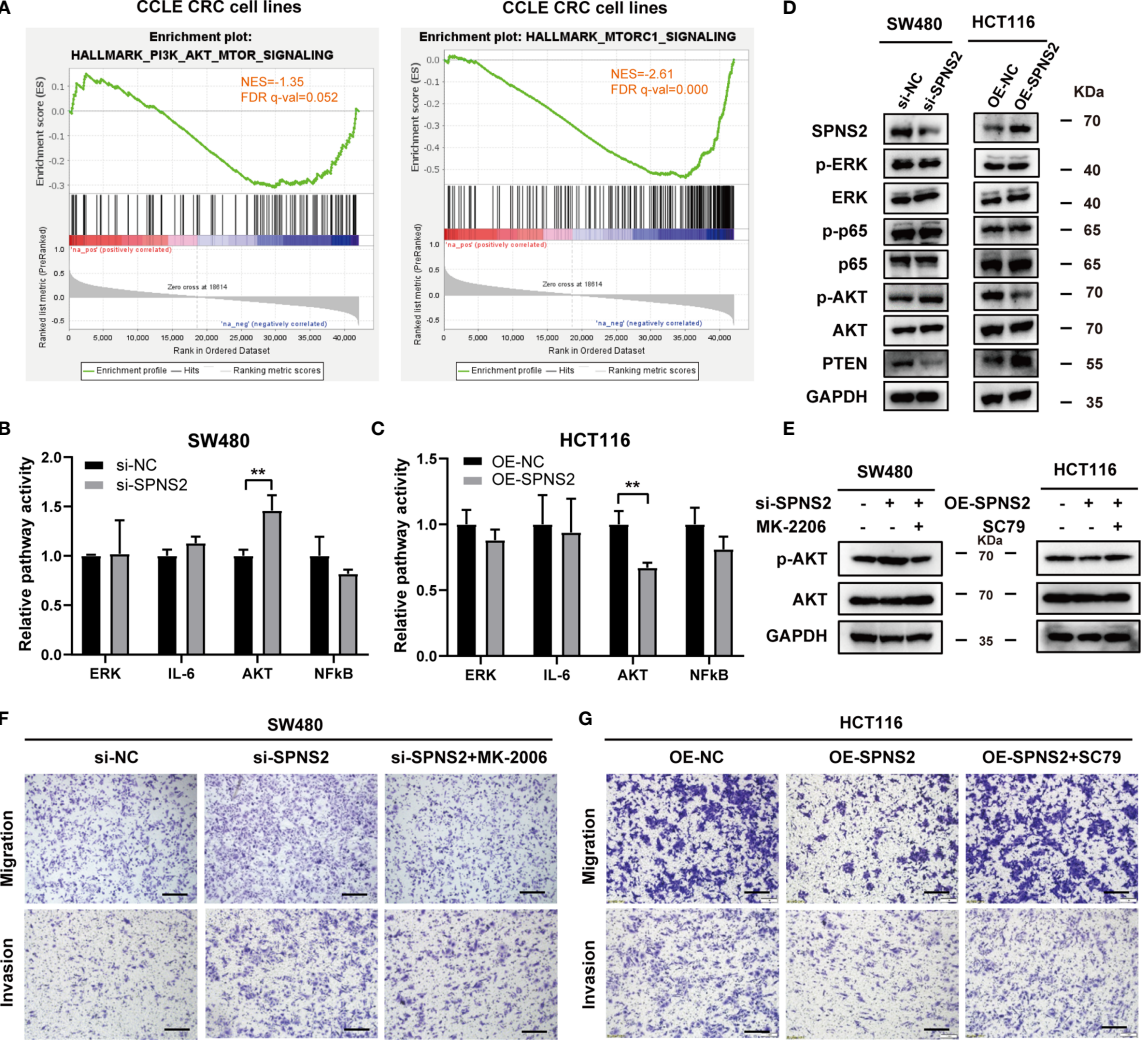
Effects of SPNS2 on the AKT signaling pathway. **(A)** GSEA analysis in CCLE CRC cell lines showed that SPNS2 expression negatively correlated with “HALLMARK PI3K AKT MTOR SIGNALING” and “HALLMARK MTORC1 SIGNALING”. **(B, C)** The relative activities (mean ± S.D.) of AKT, ERK, STAT3, and NF-κB pathways were determined *via* Qiagen reporter luciferase assay in SPNS2-overexpressing HCT116 cells or SPNS2 knockdown SW480 cells versus the NC counterparts. **p < 0.01 **(D)** A representative western blot of total AKT, phosphorylated AKT (Ser473), ERK, phosphorylated ERK, P65 and phosphorylated P65 in the indicated CRC cells. **(E)** MK2206 reduced the phosphorylation level of AKT in SW480 si-SPNS2 cells and SC79 enhanced the phosphorylation level of AKT in HCT116 OE-SPNS2 cells. **(F)** MK2206 reduced cell migration and invasion in SW480 si-SPNS2 cells (scale bar = 100 µm). **(G)** SC79 enhanced cell migration and invasion in HCT116 OE-SPNS2 cells (scale bar = 100 µm).

Given that SPNS2 can transport the intracellular S1P outside of the cell, so we speculate that SPNS2 functions through modulating the concentration of S1P between the cell and microenvironment. Indeed, the concentration of S1P in medium dramatically increased after SPNS2 overexpression in HCT116 cells by LC-MS/MS assay (**Figure S2A**). Previous researches have shown that S1P regulates the activities of numerous signaling pathways such as ERK, IL6, AKT and NF-κB (33). To elucidate the underlying molecular mechanisms of SPNS2-mediated anti-tumor effect, we screened these four cancer pathways pathway activity assay using Cignal Reporter Assay Kits of QIAGEN, which measuring the activities of downstream transcription factors through dual-luciferase format. Specifically, PI3K/Akt pathway activity is determined by measuring the luciferase activities of FoxO transcription factors, which are known downstream effectors of Akt (27). SPNS2 knockdown in SW480 cells stimulated PI3K/Akt signaling without affecting the activities of other three pathways (P < 0.01; **Figure 7B**). Conversely, Ectopic expression of SPNS2 in HCT116 cells specifically suppressed PI3K/Akt signaling (P < 0.01; **Figure 7C**). Consistently, GSEA revealed that SPNS2 expression was negatively associated with the functional gene sets of “HALLMARK PI3K AKT MTOR SIGNALING” (NES = -1.35, FDR q-value = 0.052) and “HALLMARK MTORC1 SIGNALING” (NES = -2.61, FDR q-value = 0.000) (**Figure 7A**), which is downstream signaling pathway of PI3K-AKT.

Meanwhile, examining protein level of the key component involved in these pathways by western blot further supported the results of pathway activity. Activation of NF-κB occurs depends on phosphorylation of IκB proteins. p65 subunit is one of the five members of NF‐κB family and is considered as the most potent transcriptional activator of the family (34). AKT is activated by phosphorylation on Thr308 and Ser473 (35–37). Phosphorylation of AKT at Ser473 has been reported to specifically target FOXO (38) and promote tumor progression (37, 39). PTEN is a critical upstream molecule that inhibits Akt activation in cancer (40). We found that SPNS2 overexpression in HCT116 increased PTEN expression and decreased the p-AKT (Ser473) level, while knockdown of it in SW480 cells inhibited PTEN expression and promoted AKT phosphorylation (**Figure 7D**). However, the phosphorylation levels of ERK and P65 was not altered after knockdown or overexpression of SPNS2 (**Figure 7D**). Altogether, SPNS2 inhibited the Akt activation but had no effect on the regulation of the ERK and NF-κB signaling pathways in CRC.

To further confirm the role of AKT signaling in SPNS2‐regulated migration and invasion in CRC, we performed transwell assays in SW480 si-SPNS2 and HCT116 OE-SPNS2 cells after treatments with MK2206 (an AKT inhibitor) or SC79 (an AKT activator). MK2206 inhibited the AKT phosphorylation in SW480 si-SPNS2 cells while SC79 promoted the AKT phosphorylation in HCT116 OE-SPNS2 cells (**Figure 7E**). As shown in **Figure 7F**, MK2206 reversed the promoted migration and invasion by knockdown of SPNS2 in SW480, while SC79 reversed the impaired migration and invasion by overexpression of SPNS2 in HCT116 (**Figure 7G**).

Though ectopic expression of SPNS2 promoted the release of S1P from HCT116 and increased the S1P level in the medium to ~6nM (**Figure S2A**), we found that extracellular S1P could only activate AKT signaling and enhance invasion (**Figures S2B, C**) when the concentration reached above 100nM, which was far higher than the level of S1P transported outside the cells. This indicated that the effect of SPNS2 was probably not dependent on the change of extracellular S1P concentration. Thus, SPNS2 seems to regulate pathological processes in CRC not through the S1P-dependent pathway, but possibly by other modes, which needs further investigation.

Collectively, these finding suggested that low levels of SPNS2 induced CRC cell invasion by activating PI3K-AKT signaling.

## Discussion

In the present study, we reported that the expression of SPNS2 in CRC specimens was lower than in its precursor lesion colon adenoma. And its low expression was associated with poor differentiation, advanced TNM stage and poor prognosis in CRC. Overexpressing SPNS2 suppressed CRC cell proliferation, migration, invasion, metastasis and EMT through inhibiting the AKT signaling pathway. In contrast, knockdown of SPNS2 in CRC cells promoted these phenotypes. Altogether, our study suggests that SPNS2 down-regulation may represent a crucial factor in CRC progression and be a candidate prognostic marker.

S1P, a bioactive sphingolipid metabolite that involved in many physiology and pathology process including cancer genesis and progression, is the transport target of SPNS2. It has been reported that SPNS2 generally showed a promoting effect in the genesis, apoptosis and migration of cancer, through S1P/S1PRs pathways activating downstream signaling such as AKT, STAT3, ERK, Ras and Rac (41). On the contrary, we found that SPNS2 was significantly downregulated in most types of tumors including LUAD and LUSC, which indicated the tumor suppressor function of SPNS2. Accordantly, a study demonstrated that SPNS2 induced apoptosis and suppressed survival in NSCLS cells (16). Although its expression level was significantly lower in normal tissue than in CRC and colon adenoma, CRC possessed a dramatically lower SPNS2 expression than colon adenoma, suggesting a provoking role of SPNS2 in the early stage of CRC but an inhibiting role of it during CRC progression. Indeed, we demonstrated that SPNS2 inhibited proliferation, motility and metastasis in CRC cells. More than 50% colorectal cancer are liver metastatic, which accounts for ~50% of death in colorectal cancer (42, 43) Therefore, inhibiting CRC metastasis is significant to improve clinical outcomes. Though most studies claim that SPNS2 mediates migration *via* regulating the cellular cytoskeleton (8, 44), SPNS2 may also regulate migration in other ways. EMT could enhance cell motility and invasion, and then confer metastatic properties to cancer cells (45). It is the dominant program in CRC, and promote metastasis of CRC cells (46, 47). In this study, we assessed the changes in key molecular of EMT after forced reversal of SPNS2 expression in CRC cells. SPNS2 expression was negatively associated with mesenchymal markers (Vimentin and N‐cadherin), but positively correlated with epithelial marker (E‐cadherin). Moreover, SPNS2 inhibited the expression of Slug, which is a zinc-finger transcriptional repressor of EMT. Thus, these findings suggest that SPNS2 mediates metastasis of CRC *via* inhibiting EMT and has potential to be a novel molecular target for anti-metastasis therapy in CRC.PI3K/Akt, JAK/STAT3 and ERK are reported to be simulated by SPNS2 *via* extracellular S1P/S1PRs combination in cancer cells. However, the key for S1P to exert its functions is the high concentration gradient from extracellular to intracellular, normally ~μM in plasma and ~nM in tissue (48). In the present study, after overexpressing SPNS2 in HCT116 cells, extracellular S1P concentration significantly increased without changing the intracellular S1P concentration, which was consistently with previous finds that SPNS2 does not influence the level of intracellular S1P in SPNS2 deficient mice (49) and ectopic SPNS2 expression didn’t alter intracellular level of S1P in lung cancer cells (16). But the concentration gradient of S1P did not exist in HCT116 SPNS2 overexpressing cells, because both intracellular and extracellular S1P maintained at a similar ~nM level. We also found that extracellular S1P enhanced migration and invasion only at the concentration reaching above 0.1 μM, when a high concentration gradient was built. We speculate that the function of SPNS2 in CRC was not dependent on S1P/S1PRs pathways. Actually, we revealed that inactivation of AKT pathway by PTEN was responsible for SPNS2 mediated phenotypes in CRC. The activation of the PTEN/AKT is observed in many kinds of tumor including colorectal cancer, promoting proliferation and metastasis (36). Also, the PTEN/AKT pathway and its downstream proteins play an essential role in EMT (50, 51). In this study, we observed increased the phosphorylation levels of AKT after SPNS2 knockdown, in line with a previous study in lung cancer (16). In addition, AKT inhibitors inhibited the phosphorylation levels of AKT, migration and invasion induced by knockdown of SPNS2 in CRC. Thus, SPNS2 might function by inhibiting the activation of AKT signaling pathway and then preventing EMT. However, the mechanism by which SPNS2 modulating the PTEN/AKT activity in CRC remains to be determined.

## Conclusions

In summary, our data reveal that SPNS2 acts as a tumor suppressor during CRC progression. Its expression in CRC was lower than in colon adenoma, and was regulated by DNA methylation and copy number alteration. The decreased expression of SPNS2 promoted CRC cell proliferation, migration, invasion and metastasis through activating AKT signaling pathway. We believe that our study lays the foundation for specific mechanisms of CRC progression and can contribute to the improvements for early detection and therapy for colorectal cancer.

## Data Availability Statement

The original contributions presented in the study are included in the article/**Supplementary Material**. Further inquiries can be directed to the corresponding author.

## Ethics Statement

The animal study was reviewed and approved by University of Science and Technology of China.

## Author Contributions

LL and ML conceived and designed the study. LL, YY, and FC performed the experiments. QY conducted the bioinformatics analysis. QY, LL, and ML wrote this manuscript. All authors contributed to the article and approved the submitted version.

## Funding

This work was supported by the National Natural Science Foundation of China (81602230 to LL, 81402327 to QY), the Anhui Provincial Natural Science Foundation (2008085MH288 to ML), the Fundamental Research Funds for the Central University (WK9110000025 to ML), the National Cancer Center Climbing Funds (NCC201812B036 to ML), and the Provincial Natural Science Research Project of Anhui Colleges (KJ2020A0147 to QY).

## Conflict of Interest

The authors declare that the research was conducted in the absence of any commercial or financial relationships that could be construed as a potential conflict of interest.

## Supplementary Material

Supplementary Figure 1 Correlation between SPNS2 expression and invasion-related genes expression. **(A–C)** Spearman’s correlation analysis of SPNS2 expression and CDH1 (encoding E-cadherin), CDH2 (encoding N-cadherin), VIM (encoding Vimentin) expression performed in all cell lines of CCLE. **(D–F)** Spearman’s correlation analysis of SPNS2 expression and CDH1, CDH2, VIM expression performed in CRC cell lines of CCLE. **(G, H)** Spearman’s correlation analysis of SPNS2 expression and MMP2/MMP9 expression performed in all cell lines of CCLE. (D-F) Spearman’s correlation analysis of SPNS2 expression and MMP2/MMP9 expression performed in CRC cell lines of CCLE.

Supplementary Figure 2 S1P enhances invasion and activates Akt under high concentration. **(A)** SPNS2 overexpression increased extracellular level of S1P. **(B)** Western blot analysis of phosphorylated Akt (p-Akt) and Akt in HCT116 treated with S1P at different concentrations. **(C)** The invasive ability of HCT116 cells treated with different concentration of S1P was determined by transwell assays.
