# Supplementary figures and images for: SPNS2 Downregulation Induces EMT and Promotes Colorectal Cancer Metastasis via Activating AKT Signaling Pathway
Source: Front Oncol. 2021 Jun 24;11:682773. doi: 10.3389/fonc.2021.682773 (PMC8264774; doi:10.3389/fonc.2021.682773)

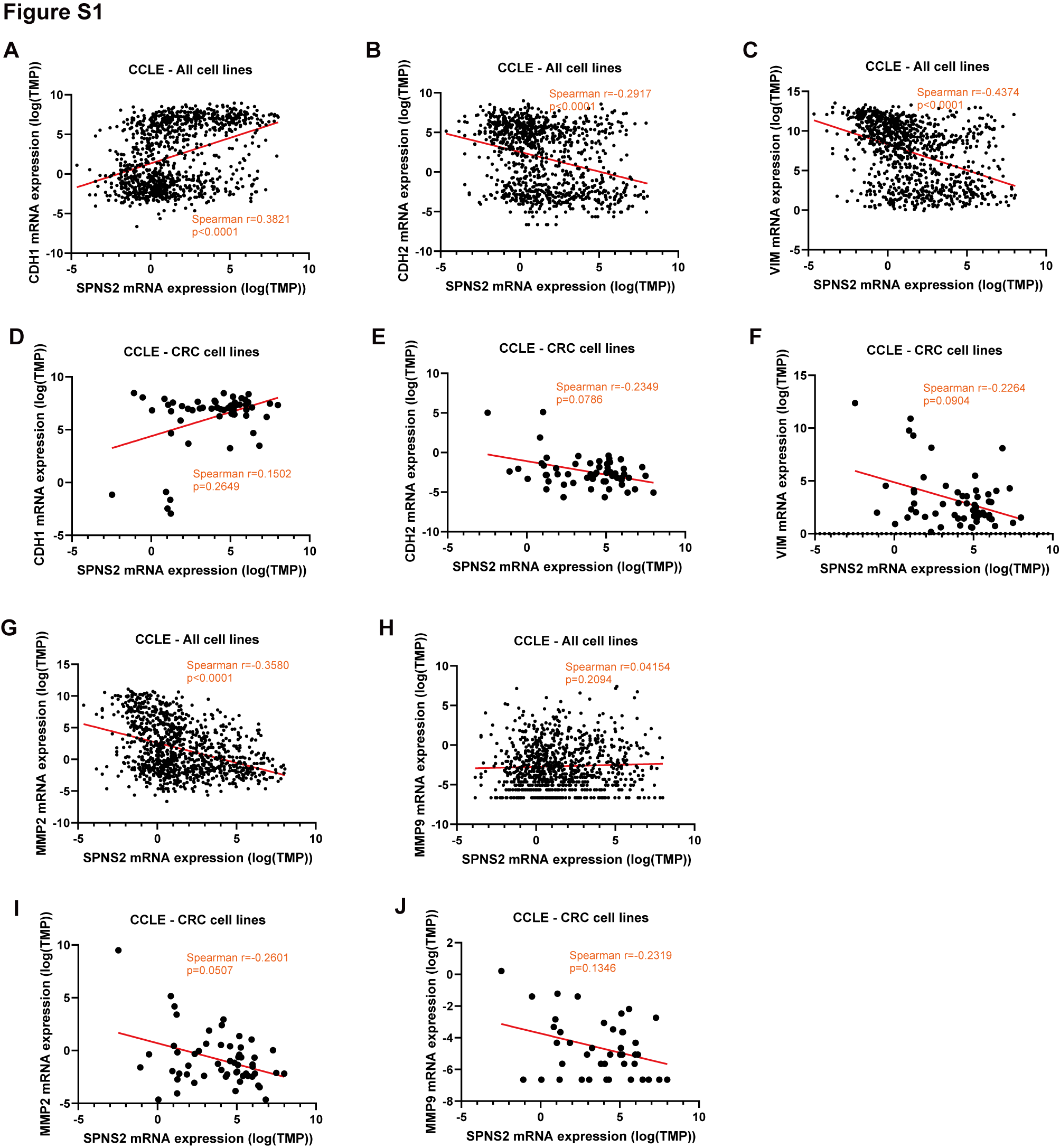

Supplement: Supplementary Figure 1 — Correlation between SPNS2 expression and invasion-related genes expression. (A–C) Spearman’s correlation analysis of SPNS2 expression and CDH1 (encoding E-cadherin), CDH2 (encoding N-cadherin), VIM (encoding Vimentin) expression performed in all cell lines of CCLE. (D–F) Spearman’s correlation analysis of SPNS2 expression and CDH1, CDH2, VIM expression performed in CRC cell lines of CCLE. (G, H) Spearman’s correlation analysis of SPNS2 expression and MMP2/MMP9 expression performed in all cell lines of CCLE. (D-F) Spearman’s correlation analysis of SPNS2 expression and MMP2/MMP9 expression performed in CRC cell lines of CCLE. [file Image_1.tif]

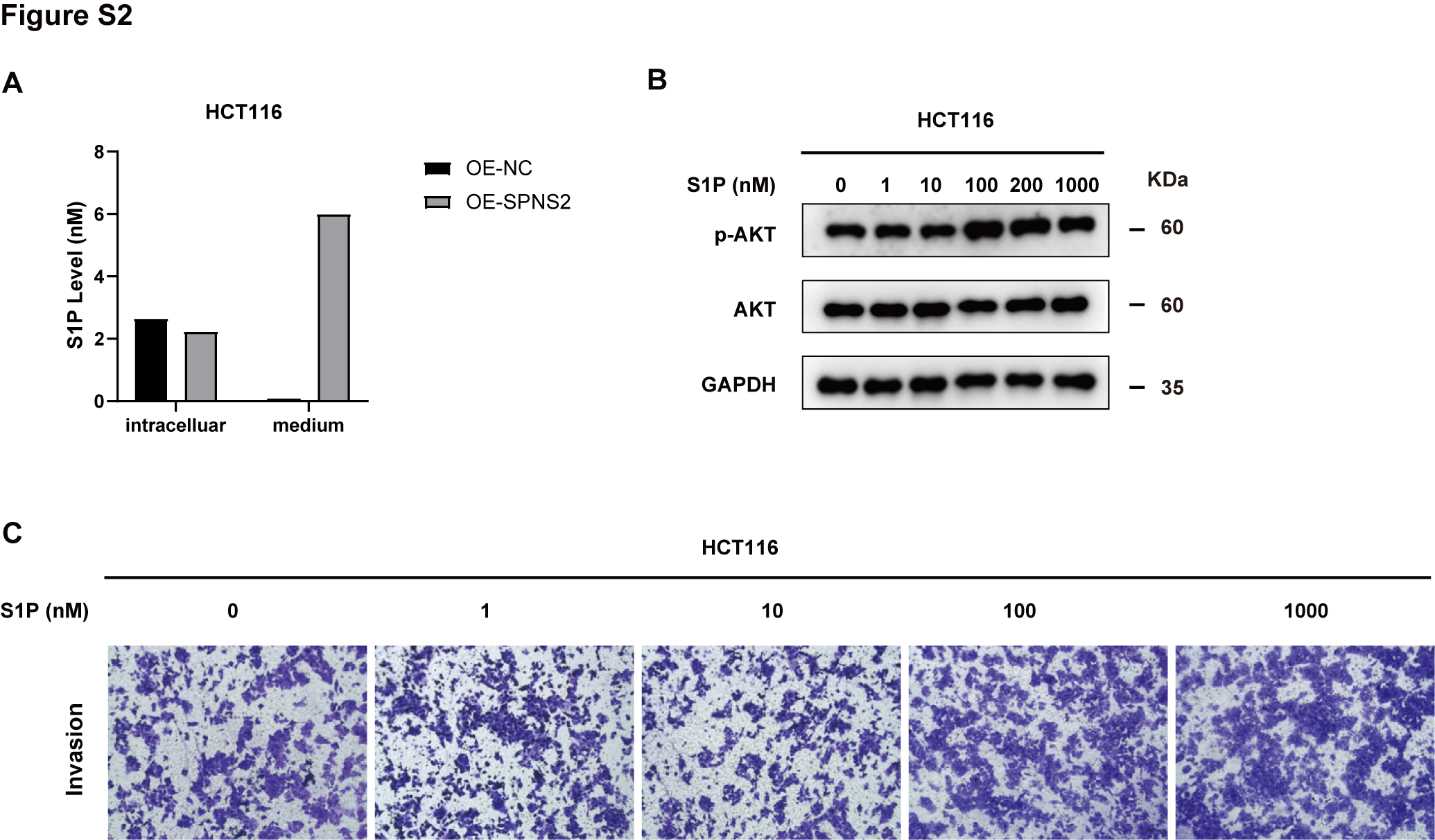

Supplement: Supplementary Figure 2 — S1P enhances invasion and activates Akt under high concentration. (A) SPNS2 overexpression increased extracellular level of S1P. (B) Western blot analysis of phosphorylated Akt (p-Akt) and Akt in HCT116 treated with S1P at different concentrations. (C) The invasive ability of HCT116 cells treated with different concentration of S1P was determined by transwell assays. [file Image_2.tif]
